# Onomatopoeia – listening to the sounds behind the words

**DOI:** 10.3205/zma001519

**Published:** 2021-11-15

**Authors:** Claudia Schlegel, Cathy Smith, Keiko Abe, Roger Kneebone

**Affiliations:** 1 Berner Bildungszentrum Pflege, Bern, Switzerland; 2 Center for Education - Division of Training & Simulation, Baycrest, Toronto, Canada; 3 Aichi Medical University, Japan; 4 Imperial College London, Faculty of Medicine, Department of Surgery & Cancer, London, UK

**Keywords:** interpersonal communication, onomatopoeia, patient's concern, interpretation

## Introduction

A patient may report to a physician: *“I tripped and heard a horrid crunch in my ankle. I cried out ow, ow, and someone came and helped me.”* “Ow” and “crunch” are examples of onomatopoeia – words that sound like the things they describe. Every language has these kinds of mimetic words, which are often used to express an impression in a personal, emotional manner and are considered indispensable in everyday conversation. Indeed, onomatopoeia can help to better explain events, give a more vivid description of emotions, and provide the receiver with a richer understanding of intent and meaning [1]. 

Onomatopoeia is a literary device like metaphor and simile in that it identifies something that is like something else. In the case of onomatopoeia, the “something” sounds like the noise made by the verbal utterance, for instance, to describe when a bottle of carbonated beverage is opened *(fizz)* or the cry of a goose *(honk)*. These “sound-like” words are also called echoic, from the mythological echo [2]. Some echoic words familiar to everyone are hush, growl, clash, gulp, screech, moan, laugh, and chortle, the latter term created by British author Lewis Carroll (1832–1898), who also gave us Alice’s Adventures in Wonderland [2]. 

As a group of simulation-based healthcare educators working in various countries, we were prompted by the context of our Japanese colleague. We learned that in Japan there are more than a thousand general mimetic words in use [3]. Our discussion made us wonder how knowledge of onomatopoeia might be applied to a patient and a clinical perspective. As pain is the most common reason for physician consultation in most countries [4], we took as our starting point an exploration of the mimetic words for pain in various cultures. 

We discovered that vocal responses related to the expression of pain seem phonologically similar between some countries, with cross-linguistic analogues such as *“ow*” in English, *“aua”* in Germany, *“ahia”* in Italy, or *“aiyo”* in Chinese [5]. These words are simple sounds that require little articulatory control while maximizing volume output, well suited to be used easily and effectively when in pain. In other countries, however, onomatopoeic words that describe pain are more nuanced and descriptive. In Japan, for example, *“gan gan”* denotes a severe headache, *“zuki zuki”* refers to back pain or to pain in several other body parts. Another colleague from Togo, West Africa, explained that various mimetic words can be used to describe different kinds of pain in his country too. He named a few examples, *“gbo gbo”* denotes headache pain, *“poupoupou”* conveys pulsating pain. Togo, like Japan, is a country where onomatopoeia is widely used and understood. 

Although the official language in Togo is French, the country is multilingual, and over 40 languages and dialects are spoken. Some patients are not sufficiently fluent in French to articulate their concerns precisely in that language. In turn, doctors and nurses cannot speak and understand all the languages and dialects spoken in Togo, so onomatopoeia plays a large part in their diagnosis. The sounds of these words cross traditional linguistic boundaries and convey meanings that are widely understood. This response resonates with the well-known Bouba/Kiki effect, where people from a wide range of cultures and languages are presented with drawings of two shapes (a softly rounded one and a jagged angular one) and asked to pair each with the made-up sounds of “bouba” or “kiki”. Almost all associate “bouba” with the rounded shape and “kiki” with the spiky one [6]. 

It is sometimes thought that onomatopoeia is the preserve of children who have not yet mastered a sophisticated vocabulary. Indeed, there are many onomatopoeia in Japanese nursery rhymes. Kasai [7] analysed all the onomatopoeia on “Kodomo-no-Uta 200” (a collection of Japanese nursery rhymes) and found how the onomatopoeia could look in children's songs in play, especially in physical form. Onomatopoeia has the power to appeal to multiple modes of sensory perception, capturing information from one context or situation that cannot be conveyed by more conventional words. Children whose vocabulary is not yet extensive have vivid imaginations, and may readily experience this world of onomatopoeia.

However, we challenge the view that onomatopoeia is intended for use by children only, arguing that the precise use of onomatopoeia for describing pain in countries such as Japan and Togo demands a different interpretation. Indeed, teaching awareness of the role of onomatopoeia in Japanese clinical settings using standardized/simulated patient (SP) methodology is an established part of the medical school curriculum [3]. SP-based scenarios are designed to include mimetic words and SPs are trained to use these words in SP/student encounters. Students are required to listen closely to the concerns of the SP and to interpret onomatopoeic words with as much precision as they would if the patient used more formal language. An example would be a female Japanese patient who comes to the doctor with severe headache. Instead using the expression severe headache, she explains that her pain started out being mild and developed to a* “gan gan”* (severe) pain. The student now has to interpret *“gan gan”* as a headache which is severe and must provide the required treatment to the patient. 

## Discussion

We suggest that onomatopoeia can be framed as an intuitive form of expression, often culturally and/or linguistically specific, through which patients communicate their concerns to healthcare providers. Carefully designed encounters with SPs, like those developed in Japan, provide excellent opportunities for trainees to practise recognizing and clarifying the meaning of these utterances. Becoming aware of the subtleties and precision of mimetic word utterances may allow clinicians to understand their patients’ needs more fully and provide appropriate treatment. When a patient’s facial cues are hidden behind a mask, such as has been the case in the recent pandemic, listening can become an even more important skill for healthcare providers to demonstrate. The pandemic notwithstanding, the increasing globalisation and movement of people between linguistic cultures means that awareness and interpretation of onomatopoeia may become an increasingly important diagnostic skill for clinicians, especially paediatricians, to master to understand what patients are trying to communicate to them. As such, simulation-based healthcare education with SPs related to patient use of onomatopoeia should be included in health professional training curricula. 

## Competing interests

The authors declare that they have no competing interests. 
